# *In vitro* selection of drought-tolerant white poplar clones based on antioxidant activities and osmoprotectant content

**DOI:** 10.3389/fpls.2023.1280794

**Published:** 2023-11-16

**Authors:** Vanja Vuksanović, Branislav Kovačević, Marko Kebert, Lazar Pavlović, Lazar Kesić, Jelena Čukanović, Saša Orlović

**Affiliations:** ^1^Department of Fruit Growing, Viticulture, Horticulture and Landscape Architecture, Faculty of Agriculture, University of Novi Sad, Novi Sad, Serbia; ^2^Institute of Lowland Forestry and Environment, University of Novi Sad, Novi Sad, Serbia

**Keywords:** abiotic stress, polyethylene glycol, tissue culture, proline, glycine betaine

## Abstract

**Introduction:**

In light of upcoming climate change, there is an urgent requirement for tree improvement regarding adaptability to drought-caused stress and the development of quick and reliable screening methodologies for genotypes’ drought tolerance. White poplar is, despite its high adaptability, considered to be an endangered tree species in Serbia, which gives it special importance in the preservation and improvement of biodiversity of riparian ecosystems. The main goal of this research was to evaluate the tolerance of five white poplar clones to the presence of polyethylene glycol (PEG 6000 molecular weight 6000) (different concentrations (e.g. 0 g/L, 1 g/L, 10 g/L, 20 g/L, and 50 g/L) in Aspen Culture Medium (ACM).

**Methods:**

The tolerance of the clones was evaluated by using morphological parameters (shoot fresh and dry weight, root fresh and dry weight), photosynthetic pigments (contents of chlorophyll a, chlorophyll b, carotenoids, and chlorophyll a+b), and biochemical parameters (total phenolic content, total flavonoid content, ferric reducing antioxidant power, antioxidant activities (DPPH activity and ABTS assay), free proline content and glycine betaine content.

**Results and Discussion:**

The values of morphological and photosynthetic pigments declined with an increase in the concentration of PEG 6000. At a concentration of 50 g/L, the content of shoot fresh mass decreased by 41%, the content of Chl a by 68%, Chl b by 65%, and Car by 76% compared to the control. Also, at the same medium, there was an increase in the content of total phenols, accumulation of proline, the content of glycine betaine as well as in antioxidant activity. Based on the obtained results, it can be assumed that more drought-tolerant clones are characterized by high values for biomass, high content of photosynthetic pigments, and high content of proline and glycine betaine in conditions similar to drought *in vitro*. Clone L-80 showed better results in most of the tested parameters, especially compared to the reference clone Villafranca.

## 1 Introduction

Drought is one of the leading abiotic factors that negatively affect the growth and productivity of plant species, which can have incalculable ecological and economic consequences (Chen and Murata, 2011). Climate change does and is expected to bring considerable problems through extreme changes in temperature and precipitation in the 21st century (IPCC, 2023). Over the past 5 years, more than half of Europe has been affected by extreme drought conditions, with major consequences for agriculture, inland waterway transport, forestry, society, and biodiversity (Ionita et al., 2022). Researchers estimated that if no climate adaptation strategies are developed and adopted in the near future, drought disruption may result in an economic loss of more than €100 billion (Mohammed et al., 2022). There is a need to reduce this risk by improving plant growth under drought-stress conditions (Ashry et al., 2022).

White poplar (*Populus alba* L.) is an indigenous species of the Republic of Serbia, which is widely used in the production of lumber, pulp, and paper, while its biomass has great potential as a source of energy. It occurs in a wide distribution range from the Mediterranean to central Asia. It is characterized by fast growth, a powerful root system, resistance to smoke and harmful gases in the air, and many other abiotic agents except prolonged flooding. However, despite its high adaptability, according to the European Forest Genetic Resources Programme (EUFORGEN) database on forest genetic resources (2003), the white poplar is classified as endangered throughout Europe. As a component of floodplain mixed forests, which are ecosystems with high biodiversity and are seriously endangered by human activity, white poplar plays a significant ecological role. In this sense, in recent years significant projects of research and preservation of variability, restoration, and reforestation with white poplars have been carried out in most European countries (EUFORGEN).

Breeding in poplars is characterized by intense evolution: from a selection of accessions from the natural stands and controlled breeding to the implementation of contemporary biotechnological methodologies (Stanton et al., 2010). Nowadays, poplar breeding programs pay more attention to tolerance to the various abiotic agents, especially drought, due to climate change, implementing advances in genomic and phenotypic tools (Rosso et al., 2023). Estimation of a plant’s tolerance to drought is a very complex process due to various interactions between drought and various physiological and biochemical phenomena that affect plant growth and development (Razmjoo et al., 2008). (Rubio et al., 2002) state that the symptoms of drought can also occur with other abiotic factors, such as salinity, and high and low temperature. In this sense, *in vitro* culture is one of the suitable models for quick assessment of the forest trees’ tolerance to abiotic agents in relatively controlled conditions opposite to all limitations in field trials caused by multiannual growth and development of tree species, such as a large area that is needed for the establishment of experiments in the field conditions, as well as the hardly controllable influence of the external multifactorial stressors present in the natural environment. The basic approach to overcoming the problem of climate change, and thus drought as a leading abiotic stressor, is precisely the selection of species and the creation of tolerant varieties. That is why it is very important to know the underlying mechanisms of tolerance of plants, especially forest woody species, to drought. As the white poplar is considered to be a model forest tree species in biotechnological research (Confalonieri et al., 2000), the development of *in vitro* drought tolerance assessment methodology is important for the improvement of testing and selection process in white poplar, but also in other forest tree species. The *in vitro* technique has been proven as an effective, cheap, and fast method in the vegetative propagation of white poplar. Also, the advantages of testing white poplar clones *in vitro* conditions compared to field trials are multiple: a greater number of repetitions, a quick response, a greater number of clones, controlled conditions, and testing can be done throughout the year. One of the main disadvantages is the fact that conditions in the field are not controlled and usually the plant is not affected only by one but a series of abiotic and biotic factors. In contrast, *in vitro*, techniques provide good opportunities for studying and understanding the effect of single stress on different physiological processes and interactions and the selection of promising clones in conditions that can be efficiently controlled. *In vitro* cultures have been used to assess drought tolerance in numerous plant species: potato (*Solanum tuberosum* L.) (Hanász et al., 2022), rice (Biswas et al., 2002), guava (*Psidium guajava* L.) (Youssef et al., 2016), blueberry (*Vaccinium corymbosum* L.) (Molnar et al., 2022), “Mexican lime” (*Citrus aurantifolia* (Christ.) Swingle) (Jafari and Shahsavar, 2022), Mexican marigold (*Tagetes minuta* L.) (Babaei et al., 2021), *Stevia rebaudiana* (Bertoni) (Hajihashemi and Sofo, 2018), *Agave salmiana* (Puente-Garza et al., 2017), Wild cherry (*Prunus avium* L.) (Vuksanović et al., 2022), olives (*Olea europaea* L.) (Silvestri et al., 2017), kiwifruit (*Actinidia chinensis* Planch.), (Wu et al., 2019), fig tree (*Ficus carica* L.) (Abdolinejad and Shekafandeh, 2022) and euramerican poplar (Popović et al., 2017). Erst and Karakulov (2023) analyzed resistance of three *Populus alba×P. bolleana* and *P. davidiana×P. bolleana* regenerants *in vitro* to osmotic stress induced by d-mannitol. Beside numerous drought tolerance studies in hydroponic (Popović et al., 2016; Ždero-Pavlović et al., 2020), greenhouse (Cocozza et al., 2016; Kebert et al., 2022a), and field (Lei et al., 2006) conditions, there is still a small amount of data on drought tolerance of forest tree species *in vitro*.

Since polyethylene glycol (PEG 6000), a neutral high-molecular weight polymer, simulates a water deficit by forming multiple hydrogen bonds with water and rendering it inaccessible to plants, it is widely used to induce osmotic stress *in vitro* (Mozafari et al., 2018; Hanász et al., 2022). Drought frequently results in oxidative stress, which modifies the redox status of plants. An imbalance between the generation of reactive oxygen species (ROS) in cells and tissues and the antioxidant defense systems that neutralize these detrimental ROS is known as oxidative stress. To overcome oxidative stress, plants evolved various defense mechanisms, including enhanced biosynthesis of compounds with strong antioxidant capacity such as polyphenols, carotenoids, proline, glycine betaine, etc. (Safaei Chaeikar et al., 2020; Babaei et al., 2021). Because of their unique structure of highly conjugated system and abundance of hydroxyl groups, particularly the 3-hydroxy groups in flavonols, phenolic compounds have a potent antioxidant effect in higher plants (Urquiaga and Leighton, 2000). Polyphenols are able to directly scavenge ROS, prevent initiation of lipid peroxidation by chelating iron ions that catalyze Fenton like reactions (Tsao, 2010). Furthermore, drought upregulates overexpression of certain transcription factors and genes (such as PFG3) that are involved in flavonoid biosynthesis (Baozhu et al., 2022). Frequently, higher amounts of accumulated flavonoids are accompanied with more tolerant species varieties, genotypes to drought stress (Selmar, 2008; Agati et al., 2012).Because of its chaperone-like activity and a key role in protecting macromolecules from oxidative stress and reactive oxygen species (ROS), free proline, as a compatible osmolyte and multifunctional amino acid, is thought to be the most important indicator of drought (Kishor et al., 2005; Szabados and Savoure, 2010). Additionally, glycine betaine is one of the quaternary ammonium compounds that plays a significant role in reducing oxidative stress and drought. Although increased generation of ROS during drought leads to deterioration and oxidation of the photosynthetic systems, chloroplast membrane, and subsequent reduction of photosynthesis, some of the ROS and RNS (reactive nitrogen species), like H2O2 and NO, are important signaling molecules, which can induce stomata closing during drought and prevent water loss and drought (Babaei et al., 2021).

Numerous studies have emphasized the importance of increased total antioxidant activity and drought tolerance in different tree plants including *Populus deltoides × Populus nigra* (Guo et al., 2010); *Citrus aurantifolia* (Christ.) Swingle) (Jafari and Shahsavar, 2022); Persian Oak and Black Poplar (Karimi et al., 2022); Oaks (Kebert et al., 2022a); *Populus przewalskii* (Lei et al., 2006); *Morus nigra* (Özelçi et al., 2022); *Populus × canadensis* (Popović et al., 2017); *Olea europaea* L. (Silvestri et al., 2017).

The research is based on the hypothesis that tolerance of examined white poplar clones on the induced drought-like conditions *in vitro* could be determined on the base of morphological parameters, photosynthetic pigments, and biochemical parameters. The main goal of this research was to study the response of white poplar clones to drought-like conditions *in vitro* to select drought-tolerant clones, and analyze the relationship between examined traits. Such studies can serve as a preliminary test for the further assessment of tolerance and adaptability of clones in field conditions.

## 2 Materials and methods

### 2.1 Plant material and micropropagation

The study involved five clones of white poplar (*Populus alba* L.). There were four Serbian clones: L-80, L-12, and LBM, selected in the natural stands, and LCM, a clone of *Populus alba* var. Bolleana, a variety popular in horticulture, as well as the well-known Italian clone Villafranca (**Table 1**). Clone Villafranca is one of the most widespread clones of white poplar in the world, characterized by the female gender, excellent rooting of hardwood cuttings, straightness of the trunk, and vigorous growth (Confalonieri et al., 2000; Vuksanović et al., 2019a; Vuksanović et al., 2019b). Thus, in this study, it is considered as a reference clone, and the reaction of other clones on examined treatments is compared with its reaction. The selected clones have exposed a high level of variability in both phenotypic and molecular evaluation (Kovačević et al., 2013). This experiment was performed in the tissue culture laboratory of the Institute for Lowland Forestry and Environment, University of Novi Sad, Serbia at the beginning of 2019. The technique of tissue culture (micropropagation by shoot tips) was applied to obtain a sufficient quantity of virus-free and genetically identical plant material in the shortest possible time. During the dormant period of the vegetation from the collection of white poplar clones of the Institute for Lowland Forestry and Environment, the culture of shoot tips was initiated based on the buds formed from apical meristem (on modified Aspen Culture Medium (ACM) with the addition of 9 g/L agar and 20 g/L sucrose, 1 µM kinetin, 1 µM BAP and 100 mg myoinositol, adjusted to pH 5.5 before autoclaving). All of the procedures of micropropagation were carried out in a sterile environment.

**Table 1:** Examined white poplar clones.

Name of clone	Origin^a)^	Description
Villafranca	Italy	Model clone, straight, narrow tree shape
L-12	Serbia	Experimental clone, vigorous straight tree shape
L-80	Serbia	Experimental clone, vigorous straight tree shape
LCM	Serbia	Horticultural clone, “Bolleana” tree shape
LBM	Serbia	Horticultural clone, straight pyramidal tree shape

Legend: ^a)^All examined clones were selected in the Institute of Lowland Forestry and Environment, Novi Sad, Serbia, except the clone “Villafranca”, which was selected at Poplar Research Institute in Casa le Monferrato, Italy.

### 2.2 *In vitro* drought treatments

After multiplication, shoots of the same age and height (about 2.0 cm) were cultivated in sterile glass jars containing 25 ml rooting medium, consisting of ACM medium, with the addition of 9 g/L agar and 20 g/L sucrose, that was supplemented with/without polyethylene glycol (PEG 6000 SIGMA™) in the following concentrations; 0 g/L (control), 1 g/L (PEG1), 10 g/L (PEG10), 20 g/L (PEG20), and 50 g/L (PEG50), adjusted to pH 5.5 before autoclaving. The range of concentrations of PEG 6000 was made based on our preliminary tests (Vuksanović, 2019), in order to find the medium that provides considerable osmotic stress, but also achieves proper agar solidification (Osmolovskaya et al., 2018, Molnar et al. (2022), and Vuksanović et al. (2022). The sterilization of the medium was performed using an autoclave at a temperature of 121°C for 20 min. For 35 days, the plantlets were allowed to develop and grow to test the clones’ tolerance to drought. The cultures were grown in controlled conditions at a temperature of t = 26 ± 2°C, under a white light of 3500 lx m^-2^ emitted by LED lamps in a 16h/8h day/night regime. The temperature was the same during the day/night regime, and the LED lamps were turned on suddenly after the night regime. The experiment was designed as completely randomized. There were 5 explants cultured in each jar. The average value of five explants at the level of one jar represented one replication. There were three jars (replications) for each tested treatment of interaction Clone × PEG concentration.

### 2.3 Measurement of morphological parameters

In this research, the following parameters were measured and expressed in grams: shoot fresh weight (SFW), shoot dry weight (SDW), root fresh weight (RFW), and root dry weight (RDW). After 35 days of cultivation on the experimental medium, the plants were removed from the jars and the fresh mass of roots and leaves was measured separately on an analytical scale. After measuring the fresh weight, the plant material was freeze-dried (lyophilized) for 72 hours at a temperature gradient from -30°C to 30°C. After drying, the dry mass of roots and leaves was measured and expressed in grams.

### 2.4 Measurement of photosynthetic pigments

For measurements of the content of photosynthetic pigments, about 0.01 g of fresh plant material was macerated in 1 mL of 96% ethanol and centrifuged at 4000 rpm for 10 min. The content of photosynthetic pigments was determined spectrophotometrically, using a MultiScan spectrophotometer (Thermo Fisher Scientific, model Multiscan GO, USA), according to (Lichtenthaler and Wellburn, 1983). Measurements included chlorophyll a (Chl a), chlorophyll b (Chl b), carotenoids (Car), and the following derived parameter chlorophyll a+b (Chl a+b). The obtained values were expressed in mg g^-1^.

### 2.5 Measurement of biochemical parameters

Ethanol extracts obtained by extracting 0.01 g of dry mass in 1 mL of 96% ethanol were used to measure biochemical parameters (excluding proline and glycine betaine).

*Total phenolic content (TPC)* was determined spectrophotometrically with Folin-Ciocalteu reagent according to the methodology proposed by (Kim et al., 2003), using gallic acid as the standard. Briefly, samples (25 mL ethanolic extract) were introduced into a microplate, and then, 125 µL of 0.1 mol/L Folin-Ciocalteu’s reagent and 100 µL of Na_2_CO_3_ 7.5% (w/v) were added. The results were expressed as mg of gallic acid equivalent (GAE) per g of dry weight (mg GAE g^-1^ DW).

*Total flavonoid content (TFC)* was measured by a spectrophotometric method as described by (Chang et al., 2002). The 30 µL of ethanolic extract was mixed with 90 µL MeOH, 6 µL 1M NaCH_3_COO, 6 µL 10% w/v AlCl_3_, and 150 µL of distilled water. The calibration curve was obtained from the absorbance of known concentrations of quercetin for the quantification of total flavonoids and the results were given as mg of quercetin equivalent per g of dry weight (mg QE g^-1^ DW).

*Ferric-reducing antioxidant power (FRAP)* was determined spectrophotometrically according to the method (Benzie and Strain, 1996). Briefly, 225 µl of FRAP reagent was added to 20 µl of ethanol extract, which was obtained by mixing acetate buffer, TPTZ solution, and FeCl_3_×H_2_O in a ratio of 10:1:1. After incubation (5 minutes) the absorbance was read at 593 nm. A standard curve was constructed using ascorbic acid (AS). The obtained results are expressed in mg AS g^-1^ DW.

*Determination of Antioxidant Activities (DPPH Activity and ABTS Assay)* The ability to neutralize ABTS (2,2′-azinobis-(3-ethylbenzothiazoline-6-sulfonic acid) radicals was determined by the method according to (Miller and Rice-Evans, 2007), while the ability to neutralize DPPH (2, 2-diphenyl-2-picrylhydrazyl) radicals was determined spectrophotometrically by the method by (Arnao, 2000). The obtained results are expressed in percentages of RSC (radical scavenger capacity). RSC or radical scavenging capacity, represents the percentage of radical species that were neutralized by antioxidants that were present in the leaf extract. The higher the RSC of the extracts, the more efficient the extract is in scavenging and neutralize radicals is, thus higher is its antioxidant potential of extract. The percentages of RSC determined by the formula:


$$\%\text{RSC}=100-({\text{A}}_{\text{A}}-{\text{A}}_{\text{B}})\text{ x }100/{\text{A}}_{\text{C}}$$

where: A_A_ - absorption of tested extract solution, A_B_ - absorption of the sample dissolved in the solvent, A_C_ - of absorption blank sample which contains only DPPH reagent and solvent.

*Free proline content (PRO)* was determined spectrophotometrically with the use of the method of (Bates et al., 1973). The reaction mixture was obtained by dissolving 20 mg of lyophilized plant material in 1 ml of 3% sulfosalicylic acid, the mixture was homogenized, then vortexed, and finally centrifuged for 10 minutes at 13000 rpm. The obtained supernatant (700 µL) was mixed with 700 µL of glacial acetic acid and 700 µL of ninhydrin solution. After an hour of incubation in a water bath (t=95°C), a colored complex was formed as a result of the reaction of proline and ninhydrin. Extraction was performed with 2 mL of toluene, and absorbance was measured at 520 nm, and the result was expressed in µmol PRO g^-1^ DW.

*Glycine betaine (GB)* was quantified by the modified spectrophotometrical method according to (Grieve and Grattan, 1983). The 10 mg of lyophilized plant material was homogenized with 500 μL of 1M H_2_SO_4_ and centrifuged at 13000 rpm for 30 minutes at 0°C. Then, 100 µl of cold KI/I_2_ was added to 250 µL of the supernatant and the mixture was left for 16 h at a temperature of 4°C. After incubation, and centrifugation at 13200 rpm, 30 minutes at 0°C, the supernatant carefully using a pipette, and triiodide crystals were dissolved in 9 ml of 1,2 - dichloroethane absorbance was read at 365 nm. The results were expressed in mg GB g^-1^DW.

### 2.6 Data analysis

The obtained results were processed by the method of two-way factorial analysis of variance, and the significance of the difference between individual PEG concentrations, clones, and their interactions was tested by Fisher’s least significant difference test (LSD test) for a significance level of p=0.05. The relationship between the investigated parameters was described using the Pearson correlation coefficient and principal component analysis (PCA). In the principal component analysis, a correlation matrix was used for data entry, and the relationship between the traits was analyzed based on their loadings with the first two principal components. The STATISTICA 13 software package (TIBCO Software Inc, 2020) was used for data processing. For visualization of data, R packages ggplot2 (Wickham, 2016) and corrplot (Wei and Simko, 2021) were used.

## 3 Results

The results of the analysis of variance indicate that the concentration of PEG had a significant effect on all examined parameters (**Table 2**). Regarding the applied concentration, the clearest differences in comparison to the control treatment were achieved on treatment PEG50, in which were also found the most distinctive differences between clones.

**Table 2:** Results of F-test on examined characters in White poplar clones^1^.

Trait	Clone (A)	PEG concentration (B)	Interaction A×B
Shoot fresh weight	3.28^*^	9.70^**^	0.65 ^ns^
Shoot dry weight	3.11^ns^	4.06^**^	0.96 ^ns^
Root fresh weight	1.69 ^ns^	3.23^**^	0.95 ^ns^
Root dry weight	1.85 ^ns^	4.28^**^	1.49 ^ns^
Chlorophyll a	5.42^**^	61.03^**^	2.08^**^
Chlorophyll b	4.14^**^	48.59^**^	1.50 ^ns^
Carotenoids	2.56^*^	27.36^**^	1.57 ^ns^
Chlorophyll a + b	5.28^**^	61.86^**^	1.91^**^
Total phenolic content	15.04^**^	4.37^**^	0.79 ^ns^
Total flavonoids content	5.15^**^	46.56^**^	1.75 ^ns^
Ferric reducing antioxidant power	28.18^**^	4.74^**^	0.81 ^ns^
ABTS radical scavenging activity	26.74^**^	14.29^**^	0.65 ^ns^
DPPH radical scavenging activity	9.98^**^	16.65^**^	1.02 ^ns^
Free proline content	256.35^**^	264.10^**^	11.87^**^
Glycine betaine	24.40^**^	97.55^**^	8.42^**^

^1^ (ns), non-significant; (*): p<0.05; (**): p<0.01.

### 3.1 Variability of morphological traits

**Figure 1:**
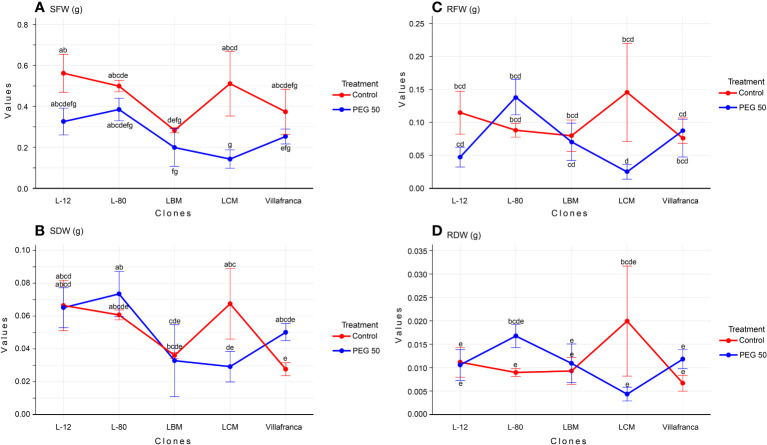
Changes in: **(A)** shoot fresh weight (SFW; g); **(B)** shoot dry weight (SDW; g); **(C)** root fresh weight (RFW; g); **(D)** root dry weight (RDW; g). Treatments: C: control (0 g/L PEG 6000); PEG50 (50 g/L PEG 6000). Differences between values of the same trait that are labeled with the same letter are not statistically significant (p>0.05). Error bars represent standard error (± SE).

In total, the values of SFW and SDW on PEG20 and PEG50 are lower than on the control treatment, while values for RFW and RDW are the highest on PEG10 (**Supplementary Table 1**). Clone L-80 stood out as the clone with the highest values of the fresh and dry mass of shoots and roots on the PEG50 medium. It had a significantly higher shoot dry weight (SDW) than LBM and LCM, but not compared to Villafranca (**Figure 1B**). In all clones, higher values of all examined morphological parameters (fresh and dry weight of shoots and roots) were observed on PEG1 compared to the control. However, this increase was not statistically significant. Compared to the control medium, only clone LCM showed a significant reduction in shoot fresh and dry weight on the PEG50 medium: SFW was reduced by 72% (**Figure 1A**), and SDW was reduced by 56% compared to the control (**Figure 1B**). There was no statistically significant difference in root fresh (**Figure 1C**) and dry weight (**Figure 1D**) between the examined clones on the PEG50.

### 3.2 Variability of photosynthetic pigments

**Figure 2:**
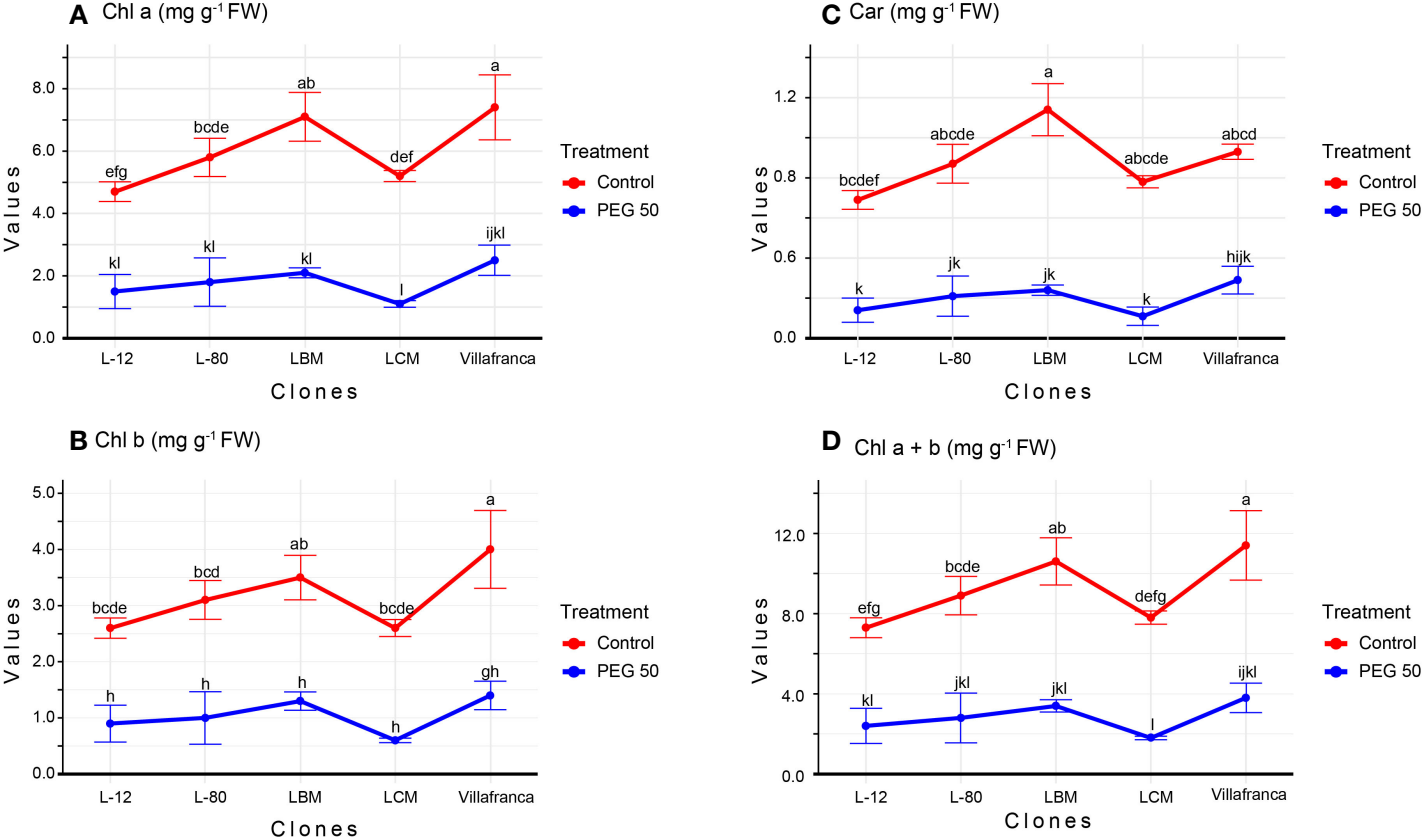
Changes in: **(A)** Chlorophyll a (Chl a; mg g^-1^ FW); **(B)** Chlorophyll b (Chl b; mg g^-1^ FW); **(C)** Carotenoide (Car; mg g^-1^ FW); **(D)** Chlorophyll a + Chlorophyll b (Chl a +b mg g^-1^ FW). Treatments: C: control (0 g/L PEG 6000); PEG50 (50 g/L PEG 6000). Differences between values of the same traits that are labeled with the same letter are not statistically significant (p>0.05). Error bars represent standard error (± SE).

An increase in PEG 6000 concentrations resulted in a decrease in photosynthetic pigments’ contents However, applied PEG6000 concentrations of 1 mg/L and 10 mg/L did not significantly affect the content of photosynthetic pigments compared to the control treatment (**Supplementary Table 1**). In most clones, all photosynthetic pigments were lower on PEG50 compared to the control (**Figure 2A**).

The examined clones showed a different response to PEG50 compared to the control. Clone Villafranca showed the lowest percentage of reduction in Chl a (66%) (**Figure 2A**), Chl b (65%) (**Figure 2B**), and Car (69%) (**Figure 2C**), while clone LCM had the highest percentage of reduction in Chl a (77%), Chl b (77%), and Car (86%). For the parameter Chl a+b on the PEG50 medium, there was no significant difference between the examined clones (**Figure 2D**).

### 3.3 Variability of biochemical traits

**Figure 3:**
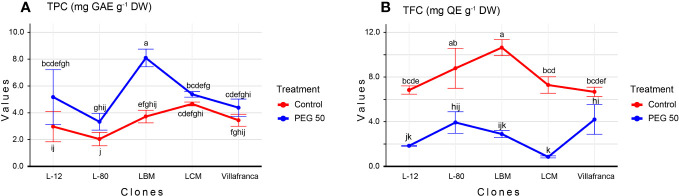
Changes in: **(A)** Total phenolic content (TPC; mg GAE g^-1^ DW); **(B)** Total flavonoid content (TFC; mg QE g^-1^ DW). Treatments: C: control (0 g/L PEG 6000); PEG50 (50 g/L PEG 6000). Differences between values of the same traits that are labeled with the same letter are not statistically significant (p>0.05). Error bars represent standard error (± SE).

The results of the analysis of variance showed that both the clones and the applied PEG concentrations had a statistically significant influence on the variation of investigated biochemical parameters. The total phenolic content (TPC) increased with increasing concentration of PEG in the media reaching 5.3 mg GAE g^-1^ DW on PEG50 (**Supplementary Table 1**). In all clones, the highest TPC was recorded in the PEG50 treatment. Clone L-80 had the lowest TPC on PEG50 treatment (3.3 mg GAE g^-1^ DW), however, this difference was not significant compared to the clone Villafranca. The highest TPC was measured in clone LBM (8.1 mg GAE g^-1^ DW) whose TPC content was statistically significantly higher than that of the reference clone Villafranca (**Figure 3A**). Unlike TPC, TFC decreased with increasing PEG concentration in the medium. The highest TFC on the PEG50 medium was measured in clone Villafranca and L-80 (4.2 and 3.9 mg QE g^-1^ DW, respectively), while the lowest TFC content was measured in clone LCM (0.8 mg QE g^-1^ DW) (**Figure 3B**).

**Figure 4:**
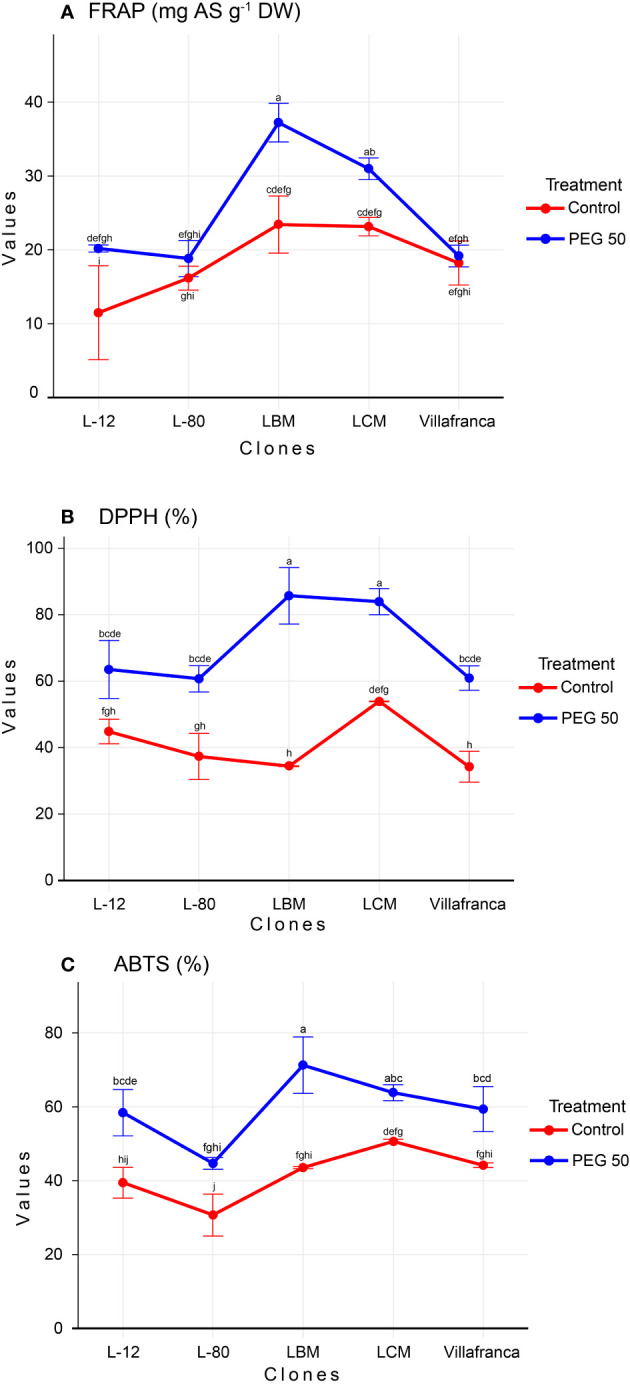
Changes in: **(A)** Ferric reducing antioxidant power (FRAP; mg AS g^-1^ DW); **(B)** 2,2′-azinobis-3-ethylbenzothiazoline-6-sulfonic acid (ABTS; %); **(C)** 2, 2-diphenyl-2-picrylhydrazyl (DPPH; %). Treatments: C: control (0 g/L PEG 6000); PEG50 (50 g/L PEG 6000). Differences between values of the same trait that are labeled with the same letter are not statistically significant (p>0.05). Error bars represent standard error (± SE).

FRAP values, as well as values of DPPH and ABTS, were increasing in a dose-dependent manner (**Supplementary Table 1**). Compared to the control treatment, in the PEG50 treatment, FRAP values increased significantly in clone L-12 by 76% reaching 20.2 mg AS g^-1^ DW, clone LBM by 59% reaching 37.2 mg AS g^-1^ DW, and clone LCM by 34% reaching 31 mg AS g^-1^ DW, while the increase in clones L-80 and Villafranca was not significant (**Figure 4A**). With the increase in stress levels, there was a significant increase in all clones in the ability of their samples to neutralize DPPH radicals compared to the control. The average value for the ability to neutralize DPPH radicals on the control was 54.4%, while the average value for neutralization on PEG50 was 70.9%., This was especially pronounced in the LBM clone where DPPH values increased by more than twice reaching 85.7% on PEG50 compared to the control where the ability to neutralize was 34.4% (**Figure 4B**).

Clone LBM (71.3%) achieved the highest ability to neutralize ABTS radicals in PEG50 treatment, while clone L-80 had the lowest ABTS radical neutralization ability (44.7%), both significantly differing from moderate performance of Villafranca (**Figure 4C**). The ability to neutralize ABTS radicals increased with increasing concentration of PEG 6000 (59.5% on PEG50) regarding control (18.5%). Also, all clones achieved a significant increase of this parameter on PEG50 treatment in comparison to the control.

**Figure 5:**
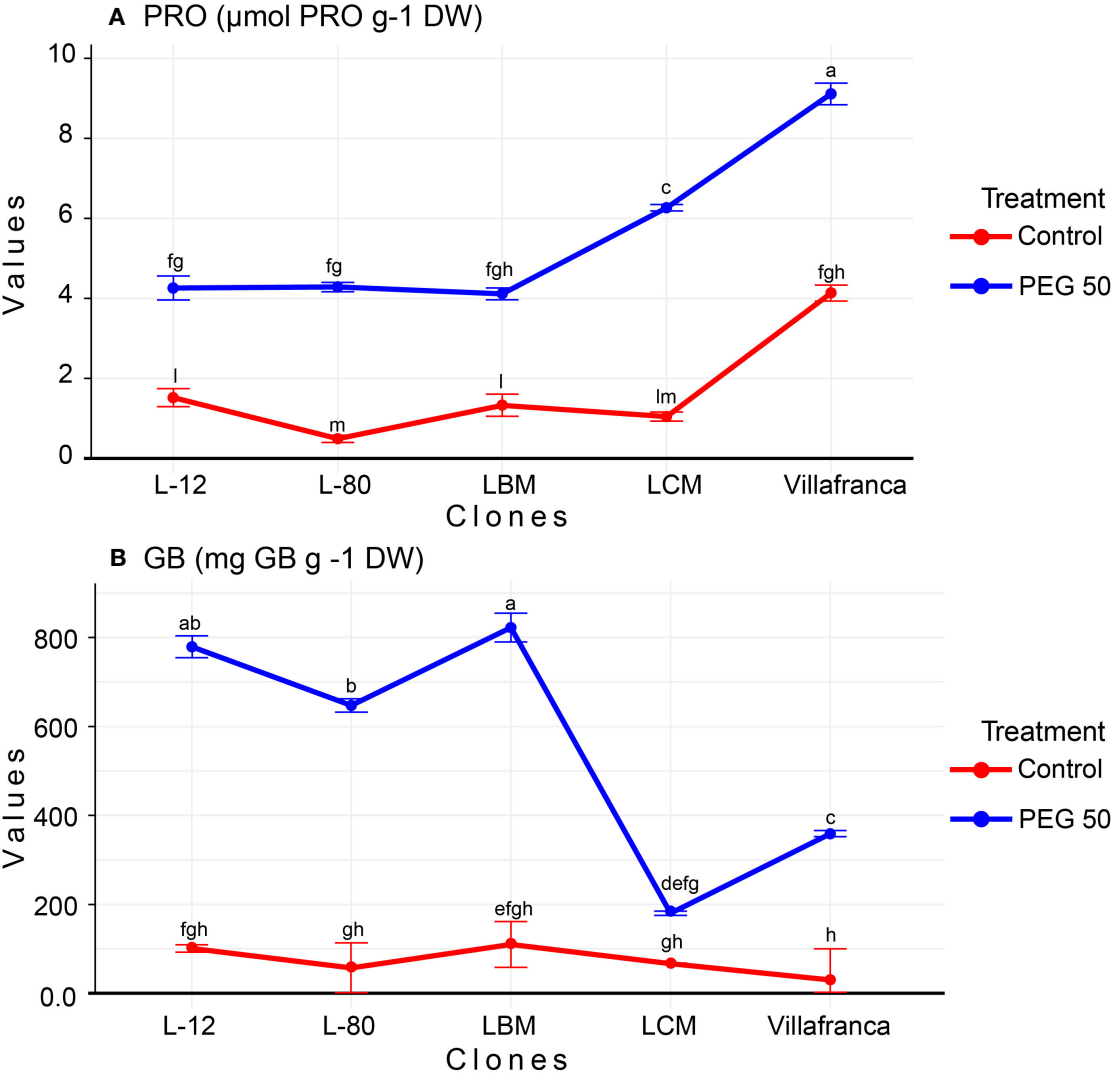
Changes in: **(A)** Free proline content (PRO; µmol PRO g^-1^ DW); **(B)** Glycine betaine (GB; mg GB g ^-1^ DW). Treatments: C: control (0 g/L PEG 6000); PEG50 (50 g/L PEG 6000). Differences between values of the same trait that are labeled with the same letter are not statistically significant (p>0.05). Error bars represent standard error (± SE).

In total, free proline content (PRO) increased as the concentration of the examined osmotic inducer increased. Thus, in the control, the average content of free proline was measured in the value of 1.7 µmol g^-1^ DW, while the average content of total proline on PEG50 was 5.6 µmol PRO g^-1^ DW (**Supplementary Table 1**). In the control treatment, the value of PRO varied among clones: from 0.5 µmol PRO g^-1^ DW for cloneL-80 to 4.10 µmol PRO g^-1^ DW for Villafranca. Also, in the PEG50 treatment, the highest free proline content was measured in the Villafranca clone (9.10 µmol PRO g^-1^ DW), while significantly lower proline content was measured in clones L-80, L-12, and LBM (**Figure 5A**). However, the greatest increase in free proline content was achieved by clone L-80, where the proline content increased by more than eight times on the PEG50 treatment (reaching 4.3 µmol PRO g^-1^ DW) compared to the control (0.5 µmol PRO g^-1^ DW). The smallest increase was recorded by clone Villafranca, where the proline content increased by only 2-fold reaching 9.1 µmol PRO g^-1^ DW compared to the control where the value was 4.1 µmol PRO g^-1^ DW.

The content of glycine betaine (GB) significantly changed depending on the concentration of PEG 6000. In total, there was a significant increase in the content of GB on PEG10 (reaching 350.5 mg GB g^-1^ DW) compared to the control (where the value was 72.8 mg GB g^-1^ DW), then it was significantly reduced on PEG20 (147.9 mg GB g^-1^ DW) compared to PEG10 treatment, while the maximum value in the content of GB plants was reached on PEG50 (reaching 557.5 mg GB g^-1^ DW) (**Supplementary Table 1**). The values for GB content ranged from 29.7 mg GB g^-1^ DW in clone Villafranca (control) up to 822.5 mg GB g^-1^ DW clone L-12 (PEG50). The clones with the greatest increase in GB content in the PEG50 treatment compared to the control were L-80 (reaching 647 mg GB g^-1^ DW) and Villafranca (reaching 358.9 mg GB g^-1^ DW), which increased their GB content by as much as 12-fold (**Figure 5B**). The smallest and non-significant increase and also the lowest GB content on PEG50 have been recorded in clone LCM (179.7 mg GB g^-1^ DW).

### 3.4 Relationship between examined parameters

**Figure 6:**
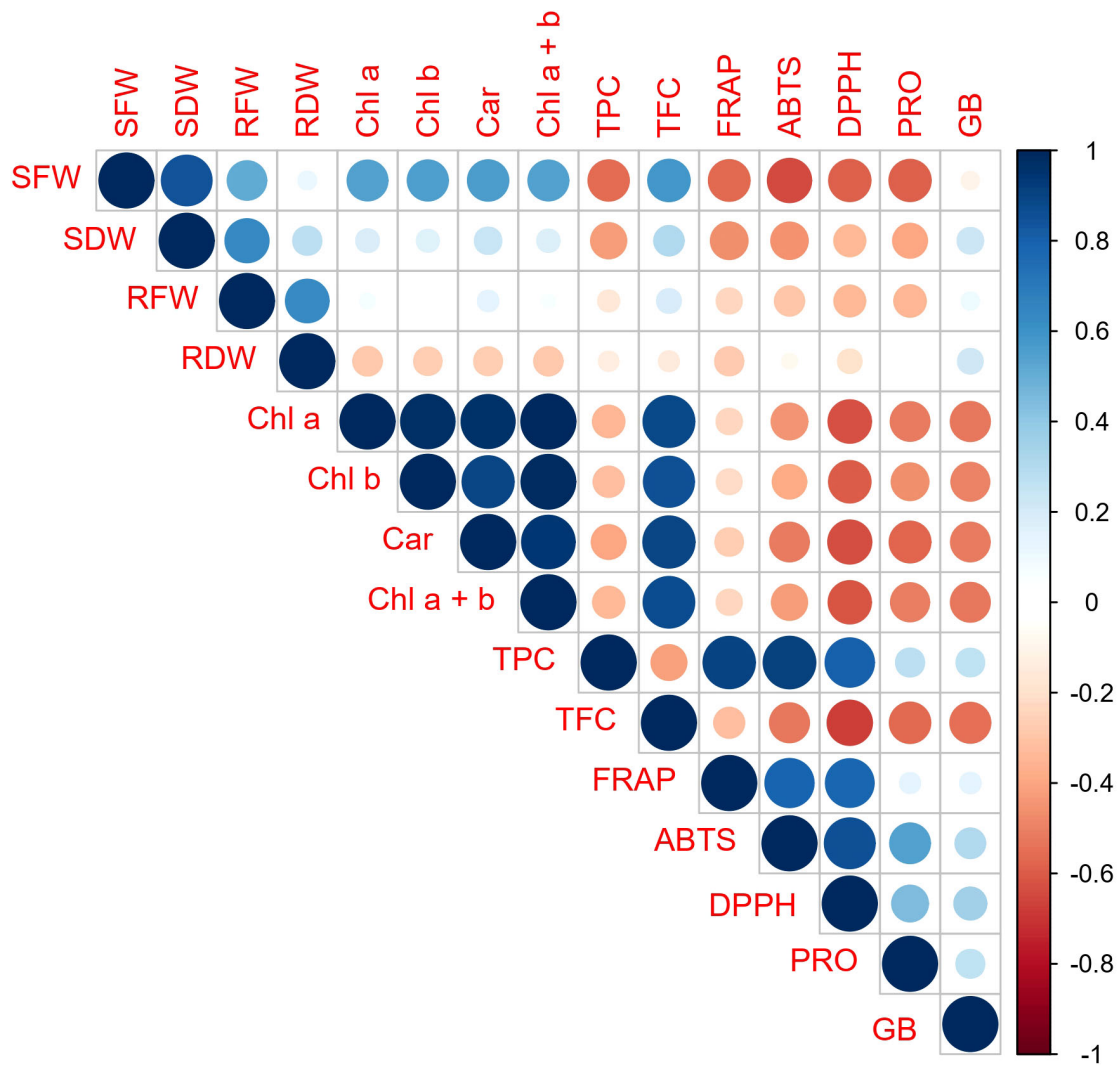
Pearson’s correlation matrix of analyzed morphological, physiological, and biochemical parameters. Abbreviations present examined parameters: shoot fresh weight (SFW); shoot dry weight (SDW); root fresh weight (RFW); root dry weight (RDW); Chlorophyll a (Chl a); Chlorophyll b (Chl b); Carotenoide (Car); Chlorophyll a + Chlorophyll b (Chl a +b); total phenolic content (TPC); total flavonoid content (TFC)); ferric reducing antioxidant power (FRAP); 2,2′-azinobis-3-ethylbenzothiazoline-6-sulfonic acid (ABTS); 2, 2-diphenyl-2-picrylhydrazyl (DPPH); free proline content (PRO); glycine betaine (GB)). Error bars represent standard error (± SE).

Based on the results of the correlation analysis (**Figure 6**), there was a positive correlation between parameters that describe the content of photosynthetic pigments, as well as between them, and TFC. On the other side, there was a positive correlation between parameters that described oxygen radical scavenging ability (ABTS, FRAP, DPPH) and TPC, and this group was in negative or poor correlation with the photosynthetic pigments’ content and TFC. The correlation of PRO and GB with the “ROS scavenging ability” group was positive, but relatively poor to moderate. However, their correlation with the contents’ of photosynthetic pigments and TFC was stronger but negative. Among morphological parameters, the strongest correlation with examined photosynthetic pigments and biochemical parameters was recorded for shoot fresh weight (SFW). The fresh mass of the shoot (SFW) was negatively correlated with most of the examined biochemical parameters (TPC, FRAP, ABTS, DPPH, and PRO). With TFC and the contents’ of photosynthetic pigments, it showed a positive correlation.

**Figure 7:**
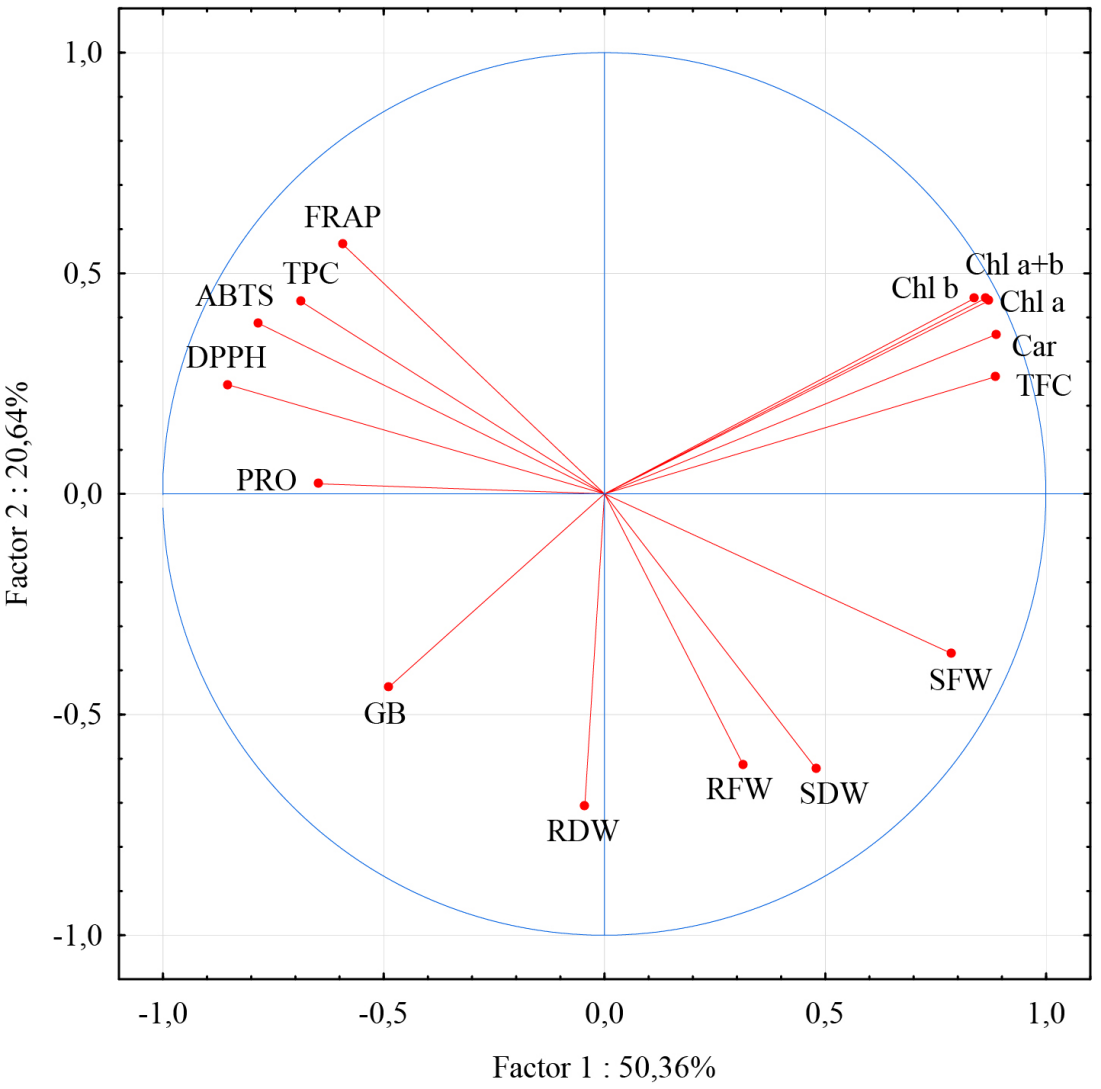
Loadings of the first two principal components for morphological physiological and biochemical parameters. Abbreviations present examined parameters: shoot fresh weight (SFW); shoot dry weight (SDW); root fresh weight (RFW); root dry weight (RDW); Chlorophyll a (Chl a); Chlorophyll b (Chl b); Carotenoide (Car); Chlorophyll a + Chlorophyll b (Chl a +b); total phenolic content (TPC); total flavonoid content (TFC); ferric reducing antioxidant power (FRAP); 2,2′-azinobis-3-ethylbenzothiazoline-6-sulfonic acid (ABTS); 2,2-diphenyl-2-picrylhydrazyl (DPPH); free proline content (PRO); glycine betaine (GB). Error bars represent standard error (± SE).

The first two principal components explained 71.0% of the total variation. According to their loadings with examined parameters, most of the parameters have their highest loadings with the first principal component. In accordance with the results of correlation analysis, this group could also be divided on contents of photosynthetic pigments TFC which were in negative correlation with ROS scavenging ability parameters and TPC. Only parameters describing the fresh and dry weight of roots and dry weight of shoots had their highest loadings with the second principal component. Relatively moderate relation with this (second) group of parameters showed the only content of betaine glycine and fresh shoot mass. Shoot fresh and dry weight achieved their strongest, although negative, correlation with parameters describing ROS scavenging ability, TPC, and PRO (**Figure 7**).

## 4 Discussion

Global climate change will undoubtedly affect the frequency and duration of droughts that may destroy forest ecosystems in the future (Salahvarzi et al., 2021). That is why an understanding of the underlying drought tolerance mechanisms of plants is of essential importance, especially in forest tree species. Studies on the tolerance of white poplar to various abiotic and biotic factors have been conducted, such as: tolerance to acidity stress (Vuksanović et al., 2019d); salinity (Sixto, 2005; Vuksanović et al., 2019c; Zhang et al., 2019), drought (Yang et al., 2016), heavy metals (Kebert et al., 2017; Kovačević et al., 2020; Kebert et al., 2022b; Katanić et al., 2015; Fischerová et al., 2006). Polyethylene glycol has several advantages (Osmolovskaya et al., 2018) and allows a convenient way to assess the effects of drought on plant growth and development in controlled conditions (Vuksanović et al., 2022). In the present study, five white poplar clones were examined for their drought tolerance *in vitro* and different clones were exposed to four different concentration ranges of PEG 6000 and control, used to induce drought-like stress conditions, had their potential drought tolerance examined *in vitro*. This research was focused on tracking poplar clones’ responses at morphological, photosynthetic, and biochemical level. Generally, most of the examined parameters decreased in a dose-dependent manner, except for TFC, root biomass traits, and GB. The treatment PEG50 provided the most intense drought-like stress effect and achieved the most clear distinction between examined white poplar clones, which was proposed it for further *in vitro* drought tolerance studies.

### 4.1 Morphological traits

**Figure 8:**
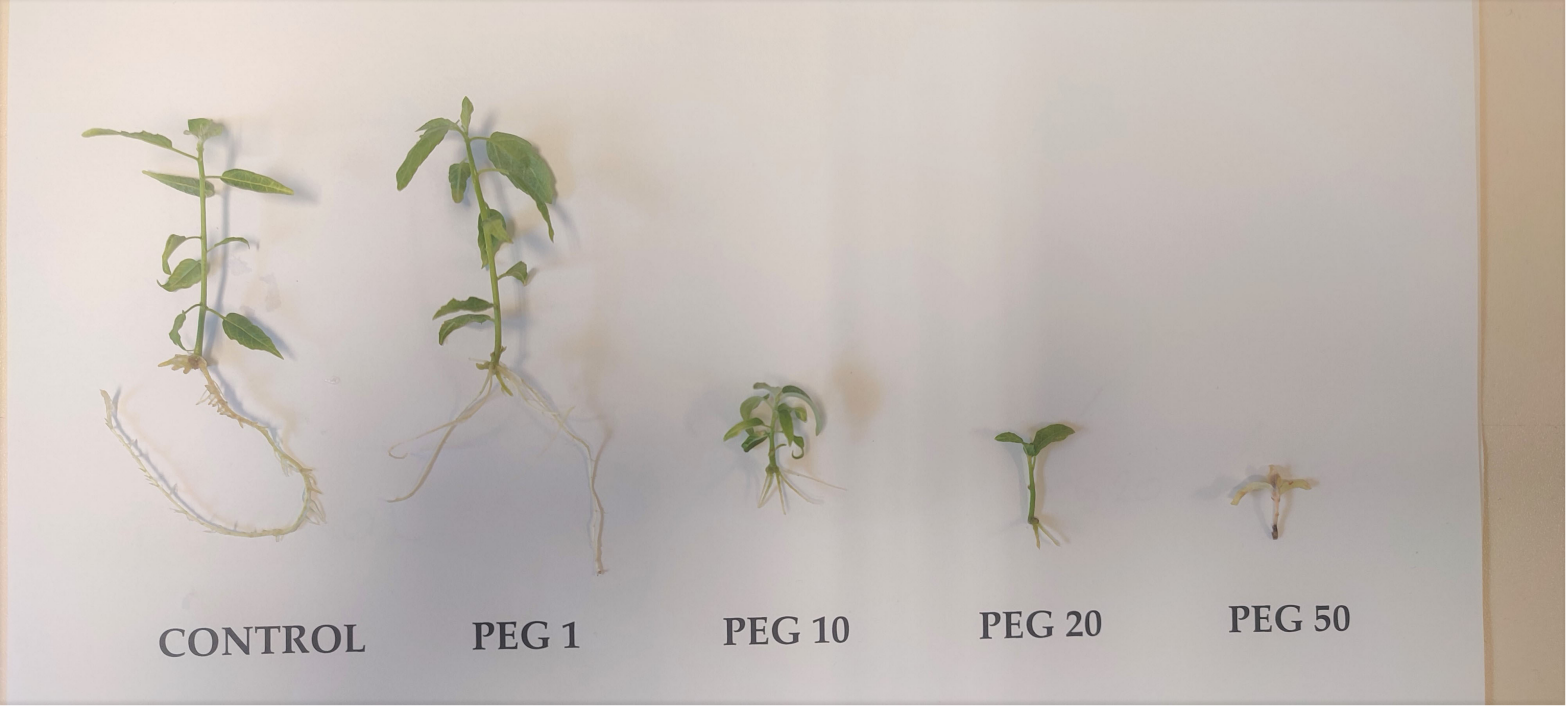
Explants of clone L-12 after cultivation on examined PEG concentrations. (control): 0; (PEG1): 1 g/L PEG 6000; (PEG10): 10 g/L PEG 6000; (PEG20): 20 g/L PEG 6000; (PEG50): 50g/L PEG 6000. Error bars represent standard error (± SE).

Based on the obtained results, it can be concluded that high PEG concentrations induced a decrease in shoot and root weight accumulation (**Figure 8**). The reduction of fresh and dry weight under the influence of polyethylene glycol was also recorded in other plant species. For example, (Akte et al., 2011) examining the tolerance of rice *in vitro*, found that the dry and fresh mass was significantly reduced when PEG was added to the MS medium at a concentration of 4%. Compared to the control, the significantly lower fresh weight of *Stevia rebaudiana* leaves was also measured by (Hajihashemi and Sofo, 2018) in the treatment with PEG 15% (w/v). Also, (Jaafar et al., 2021) performed a screening of 12 lines of cotton and observed that shoot and root weight decreased with an increase in PEG concentration from 0 to 15%. (Zhong et al., 2018), also found that fresh shoot weight decreased with increasing concentration of PEG 8000 in kiwifruit (*Actinidia* sp.) Therefore, it could be assumed that the drop in the fresh weight of the plant under the influence of drought is a consequence of the reduced growth of the plants as well as the limited capacity of the roots to provide water to the leaves. The results of this analysis suggest that although the difference was not statistically significant, it seems that the fresh and dry mass of the roots tend to increase in clone L-80 as the concentration of PEG in the medium increased. An increase in dry matter *in vitro* seeds was also obtained in tomato tolerance tests *in vitro* by adding PEG in the concentration range of 0-295 g/L (Aazami et al., 2010). Given that the root has an important role in the uptake of nutrients and water, it can be assumed that clones tolerant to drought showed a greater mass of roots compared to less tolerant ones. This could be explained by faster osmotic adjustment and retention of a larger amount of water in the roots.

If we consider the shoot’s fresh and dry mass as an ultimate result of the ability of the plant to cope with the drought stress, it is important to relate these parameters with the examined photosynthetic pigments and biochemical parameters to evaluate their significance. Due to their close relationship with the response of the plant to disturbances of the oxidative and osmotic status of the cell, they could provide information on the importance of the regulation of these characteristics of the cell’s metabolism in the mitigation of drought stress. In this study, a close negative relationship is found between shoot fresh and dry mass and some biochemical parameters, namely: FRAP, ABTS, DPPH, and TPC, but relatively moderate positive with photosynthetic pigments and TFC (**Figures 6**, **7**). The clear negative correlation of shoot mass with and parameters of ROS scavenging ability suggest that those clones that considerably increased production of antioxidant substances to cope with disturbances of oxidative status related to drought conditions could be considered as less tolerant. These results are consistence with the results of (Vuksanović et al., 2019c) who studied the reaction of white poplar clones on rooting media differing in NaCl concentration. However, in work dealing with wild cherry clones on multiplication media differing in PEG concentration by (Vuksanović et al., 2022), TPC appeared to be in positive correlation with morphometric parameters that described shoot number and height, while FRAP and ABTS achieved with those morphological parameters poor correlation. Also, FRAP, DPPH, ABTS, as well as TPC, and TFC were not correlated with shoot fresh and dry mass in the study of (Vuksanović et al., 2019d) dealing with the reaction of white poplar clones on rooting media differing in pH from pH 3.0 to pH 5.5, suggesting that this topic needs further studies.

### 4.2 Photosynthetic pigments

A large embodiment of literature demonstrated that drought conditions induce a reduction of photosynthetic pigments’ content and thus a decrease in the intensity of photosynthesis (Jaleel et al., 2009; Xia et al., 2022). In this study, the contents of all examined photosynthetic pigments were in strong positive correlation (**Figures 6**, **7**), suggesting that the reaction of clones on examined treatments was similar by all of them. All examined white poplar clones significantly reduced the content of all photosynthetic pigments on PEG50 compared to the control. (Lei et al., 2006), in their work on poplar (*Populus przewalskii*), showed that the reduced content of photosynthetic pigments was a consequence of the increased activity of enzymes (superoxide dismutase (SOD), guaiacol peroxidase (GPX), and glutathione reductase (GR)) enrolled in degradation of chlorophyll under drought conditions. Similar results were obtained by (Guo et al., 2010), investigating the tolerance of hybrid poplar *Populus deltoides × Populus nigra* clones, where there was also reduced chlorophyll content due to different regimes of limited water availability. A decrease in the content of photosynthetic pigments with an increase in PEG concentration was also recorded in other plant species. Thus, (Ghassemi-Golezani et al., 2018), observed reduced total chlorophyll and carotenoids content upon treatment with 16 mM PEG *in vitro* in *Allium hirtifolium*, while (Hussein et al., 2017), reported that total chlorophyll content was significantly reduced in strawberries treated with 2% PEG *in vitro*. Additionally, a reduction in total chlorophyll content due to drought-induced by PEG has also been documented in *Stevia rebaudiana* under treatment with PEG 15% (w/v) (Hajihashemi and Sofo, 2018) and in peanuts (*Arachis hypogaea*) under treatment with PEG-6000; 20% (w/v) (Shivakrishna et al., 2018). In this study, clones L-80 and L-12 stood out as they achieved the lowest percentage reduction in pigment content under PEG50 treatment, almost twice lower than clone Villafranca. This suggests their good tolerance against examined drought-like *in vitro* conditions, particularly in comparison with clone Villafranca. A decrease in the content of Chl a, Chl b, and Car was recorded by (Razavizadeh et al., 2019), who investigated the influence of different concentrations (2, 4, 6, and 8% (v/w)) of PEG 6000 on the tolerance of *Thymus vulgaris* L. (thyme) *in vitro*. Also, (Mehmandar et al., 2023), in their investigation of the responses of three Iranian melon genotypes to PEG (0.009, 0.012, and 0.015 M) state that there was a decrease in Chl a, Chl b, and Car content when using this osmolyte. Interestingly, (Radi et al., 2023), investigated the influence of PEG concentrations (0, 50, 100, and 150 g/L) on cactus pear (*Opuntia ficus indica*) and reported that the highest chlorophyll b content was recorded in shoots grown for 3 weeks on MS medium containing 150 g/L PEG.

### 4.3 Biochemical traits

#### 4.3.1 Antioxidant activities

Previous research showed that under the influence of drought, there were numerous changes in plant metabolism (Jaleel et al., 2009). This study showed a high accumulation of total phenols in poplar genotypes under the influence of PEG 6000. The total phenolic content increased by 56% on PEG50 compared to the control. Phenolics as secondary metabolites play an important role in plants’ protection against diseases, pests, and adverse environmental conditions including drought (Li et al., 2011). Increased phenolic content under the influence of stress caused by polyethylene glycol was also obtained in other plant species: *Thymus vulgaris* L. (6% PEG 6000) (Razavizadeh et al., 2019) *Morus nigra* cv. ‘Eksi Kara’ (355 g/L PEG 8000) (Özelçi et al., 2022); *Stevia rebaudiana* (4% PEG 6000) (Ahmad et al., 2020); *Allium hirtifolium* (16 mM) (Ghassemi-Golezani et al., 2018).

In this research, the concentration of PEG 6000 of 50 g/L led, in total, to a decrease in the content of total flavonoids by 63% compared to the control (**Supplementary Table 1**). Clones L-80, Villafranca, and L-12 achieved the highest content of flavonoids on PEG50. Furthermore, it can be assumed that the clones that exhibited the lowest flavonoid content are the least tolerant to drought stress caused by the addition of PEG 6000. Given that clone L-80 accumulated the highest amounts of flavonoids on PEG50, it can be assumed that it is the most tolerant clone according to this parameter. The TPC and TFC differed in the correlation with other parameters: TPC was positively correlated with FRAP, ABTS, and DPPH, and negatively with all examined photosynthetic pigments, while the opposite was in the case of TFC. Also, although both deceased on PEG50, the correlation between them was weak, indicating a clone specificity in their reaction to drought-like conditions with these two parameters. The clone L-80 achieved the lowest values of TPC on PEG 50, but not significantly lower than Villafranca, suggesting its good performance according to this parameter.

The ABTS, DPPH, and FRAP inhibition activities are the most commonly used assays based on electron transfer mechanisms to assess antioxidant capacity during drought stress. The obtained research results show a strong positive correlation between them and that the examined clones significantly increased the antioxidant activity (ABTS, DPPH, FRAP) on PEG50. A strong positive correlation among biochemical assays based on electron transfer mechanisms has been thoroughly explained (Huang et al., 2005). Increased antioxidant activity under drought stress conditions was previously reported in *Agave salmiana* exposed to 30% PEG; (Sarker and Oba, 2018), as well as in *Amaranthus tricolor* where plants were exposed to drought induced by reduced watering regimen and water field capacity (FC) drought from 0 to 30% FC, but also in the wild cherry *in vitro* after medium supplementation with 50 g/L of PEG (Vuksanović et al., 2022), Furthermore, increasing patterns of antioxidant power were noted in *Quercus robur* and *Quercus cerris* exposed to drought stress (Kebert et al., 2022a) However, some authors found that drought induced by 100 and 200 mOsm PEG 6000 in hydroponic culture caused significant decrease of DPPH and FRAP values in hybrid black poplar (Popović et al., 2017). Interestingly, clones L-80 and Villafranca for that the positive reaction by shoot and root dry mass, as well as clones L-12 and LCM for that negative reaction by the same parameters was recorded on PEG50 compared to control treatment, achieved relatively moderate increment of antioxidant capacity, considerably weaker than clone LBM. This clone showed no significant difference between two treatments by biomass parameters, but considerable difference in examined parameters of antioxidant capacity. Relatively moderate increment of antioxidant capacity was also recorded in white poplar clones tolerant to salty (100 mM NaCl) (Vuksanović et al., 2019c) and acidic (pH 3) (Vuksanović et al., 2019d) medium.

#### 4.3.2 Clone-specific osmolyte (glycine betaine and proline) variability

A significant amount of literature has been devoted to elucidating how the increased content of various osmolytes, such as proline or glycine betaine, is related to the increased stress tolerance to drought in plants (Kebert et al., 2023). Proline is a multifunctional amino acid and a crucial marker of abiotic stress that acts as an antioxidant and ROS quencher involved in the control of redox balance, osmotic pressure, energy status, nutrient availability, photosynthesis, and mitochondrial respiration as well as serving as a signaling molecule that modifies gene expression (Szabados and Savoure, 2010; Alvarez et al., 2022). In our study, all examined PEG 6000-treated clones showed a clearly increased level of free proline when compared to controls, with clones LCM and Villafranca standing out the most. A similar increasing trend of free proline was also reported in vitro in other plant species such as Prunus dulcis (Mill.) when it was treated with 7.5% PEG 6000 in MS medium (Karimi et al., 2022) as well as in Tacca leontopetaloides (Martin et al., 2021). Other plant species, including rice (Akte et al., 2011), almond (Akbarpour et al., 2017), mango (Pradhan et al., 2021), potato (Kumar et al., 2020), and Fragaria ananasa (Hussein et al., 2017), have also shown a drought-induced increase in free proline levels. In addition, greenhouse pot experiment under drought conditions with woody plant species such as pedunculate oak (Kebert et al., 2023), Persian oak (Quercus brantii Lindl.) and European black poplar (Populus nigra L.) also exhibited increased proline content (Karimi et al., 2022). Increased proline levels may be linked to improved plant tolerance to various abiotic stresses, including drought, because of the strong positive correlation between proline and antioxidant potential (FRAP and ABTS) that was observed in this study. Another explanation for elevated levels of proline may be related to the fact that drought alters the metabolism of proline by upregulating the proline biosynthetic gene P5CS1 and downregulating the proline catabolic gene ProDH (Yoshiba et al., 1997; Alvarez et al., 2022). Regarding quaternary nitrogen compounds, on the other hand, it was discovered that all inspected poplar clones were affected by the drought and that there was a high level of variation among them in terms of the response to the drought-induced glycine betaine response. All clones exhibited higher amounts of GB compared to untreated controls, but the clones L-12 and LBM were the most prominent in terms of GB levels under drought. Intriguingly, the PEG-treated clones LCM and Villafranca that showed the greatest increases in proline levels also showed the lowest increases in GB levels compared to non-treated controls. This might indicate the clone-specificity, where the same PEG50 treatment in some clones induces a proline response (as it did in LCM and Villafranca) while in other clones (LBM, L-80, and L-12) it induced a more dramatic GB response. Given that both metabolites utilize the same available nitrogen sources for their biosynthesis, this reciprocity between glycine betaine and proline is already known (Carrilo et al., 2008; Carrilo, 2018). The duration of the stress also affects whether the nitrogen is directed to proline or GB biosynthesis, as proline typically responds more quickly to short-term stresses while GB biosynthesis is temporally delayed in comparison to other significant osmolytes, such as proline (Carrilo et al., 2008; Woodrow et al., 2017; Carrilo, 2018). Similarly, drought-induced increasing patterns of GB were also recorded in pedunculate oak (Kebert et al., 2023), vanilla (Martínez-Santos et al., 2021), and sugar cane (Hernández-Pérez et al., 2021). In addition, increased content of glycine betaine under drought stress (induced by PEG; 50–150 g/L) was recently reported in the cactus in vitro (Radi et al., 2023). This increment in GB may be attributed to enhanced drought tolerance due to the high osmoprotective properties of GB since it is also known that GB can activate various antioxidant enzymes such as peroxidases, catalase, and SOD (Chen and Murata, 2008). Finally, GB can serve as a stabilizer and chaperone to photosynthetic proteins including oxygen-evolving PSII complex, and protect them from oxidative damage (Ashraf and Foolad, 2007).

## 5 Conclusions

The use of biochemical tests *in vitro* research is of great importance considering the rare implementation of these tests in research on the tolerance of woody species to abiotic factors. There was a negative correlation of shoot mass with and parameters of ROS scavenging ability, suggesting low drought tolerance of clones with high production of antioxidant substances. There was clear of agglomeration of examined clones to those that accumulated proline and those that accumulated glycine betaine as a response to drought-like conditions. We assume that clone L-80 is more tolerant to drought than the other examined white poplar clones, but not considerably better than the reference clone Villafranca. Namely, in conditions similar to drought *in vitro*, this clone achieved high values for biomass and content of photosynthetic pigments, but moderate values of parameters describing ROS scavenging ability. At the same time it accumulated a low content of proline and a high content of glycine betaine. On the other side, clone LCM could be regarded as the most susceptible, because of its relatively strong decrease in biomass, low content of photosynthetic pigments, and low values of TFC and GB, along with high values of TPC, FRAP, ABTS, DPPH, and PRO in examined drought-like conditions. According to the results of the study, medium with 50 g/L PEG could be proposed for further studies on variability of the responses of white poplar clones to drought stress *in vitro*. Also, examined biochemical parameters proved to be indicative in the selection of drought-tolerant clones, but should be further studied in future research on drought tolerance in white poplar.

## Data availability statement

The original contributions presented in the study are included in the article/**Supplementary Material**. Further inquiries can be directed to the corresponding author.

## Author contributions

VV: Conceptualization, Data curation, Formal Analysis, Investigation, Methodology, Project administration, Software, Validation, Visualization, Writing – original draft, Writing – review & editing. BK: Conceptualization, Data curation, Formal Analysis, Investigation, Methodology, Software, Supervision, Validation, Visualization, Writing – original draft, Writing – review & editing. MK: Conceptualization, Data curation, Investigation, Methodology, Software, Supervision, Validation, Visualization, Writing – original draft, Writing – review & editing. LP: Data curation, Software, Visualization, Writing – original draft, Writing – review & editing. LK: Validation, Writing – original draft, Writing – review & editing. JC: Writing – original draft, Writing – review & editing. SO: Conceptualization, Funding acquisition, Project administration, Resources, Supervision, Writing – original draft, Writing – review & editing.
